# Using CHADS_2_, R_2_CHADS_2_, CHA_2_DS_2_-VASc score for mortality prediction in patients with abnormal low and high ankle-brachial index

**DOI:** 10.7150/ijms.49018

**Published:** 2021-01-01

**Authors:** Nai-Yu Chi, Ho-Ming Su, Wen-Hsien Lee, Wei-Chung Tsai, Ying-Chih Chen, Tzu-Chieh Lin, Ye-Hsu Lu, Chee-Siong Lee, Tsung-Hsien Lin, Wen-Ter Lai, Sheng-Hsiung Sheu, Po-Chao Hsu

**Affiliations:** 1 Division of Cardiology, Department of Internal Medicine, Kaohsiung Medical University Hospital, Kaohsiung Medical University, Kaohsiung, Taiwan.; 2 Faculty of Medicine, College of Medicine, Kaohsiung Medical University, Kaohsiung, Taiwan.; 3 Department of Internal Medicine, Kaohsiung Municipal Siaogang Hospital, Kaohsiung, Taiwan.; 4 Department of Internal Medicine, Kaohsiung Municipal Ta-Tung Hospital, Kaohsiung, Taiwan.

**Keywords:** all-cause mortality, cardiovascular mortality, CHADS_2_ score, CHA_2_DS_2_-VASc score, chronic kidney disease, R_2_CHADS_2_ score

## Abstract

Abnormal low and high ankle brachial index (ABI) is regarded as peripheral artery disease (PAD) which has extremely high morbidity and mortality. How to identify high-risk PAD patients with increased mortality is very important to improve the outcome. CHADS_2_, R_2_CHADS_2_, and CHA_2_DS_2_-VASc score are clinically useful scores to evaluate the annual risk of stroke in patients with atrial fibrillation. However, there was no literature discussing the usefulness of these scores for cardiovascular (CV) and all-cause mortality prediction in the patients with abnormal ABI. This longitudinal study enrolled 195 patients with abnormal low (< 0.9) and high ABI (> 1.3). CHADS_2_, R_2_CHADS_2_, and CHA_2_DS_2_-VASc score were calculated for each patient. CV and all-cause mortality data were collected for outcome prediction. The median follow-up to mortality was 90 months. After multivariate analysis, CHADS_2_, R_2_CHADS_2_, and CHA_2_DS_2_-VASc score were significant predictors of CV and all-cause mortality (all *P* < 0.001). CHA_2_DS_2_-VASc score had a better additive predictive value than CHADS_2_ and R_2_CHADS_2_ score for CV mortality prediction. R_2_CHADS_2_ and CHA_2_DS_2_-VASc score had better additive predictive values than CHADS_2_ score for all-cause mortality prediction. In conclusion, our study is the first study to investigate the usefulness of CHADS_2_, R_2_CHADS_2_, and CHA_2_DS_2_-VASc score for mortality prediction in patients with abnormal ABI. Our study showed all three scores are significant predictors for CV and all-cause mortality although there are some differences between the scores. Therefore, using the three scoring systems may help physicians to identify the high-risk PAD patients with increased mortality.

## Introduction

Ankle-brachial index (ABI) is a simple and reliable diagnostic tool for peripheral arterial disease (PAD) 1,2. ABI of each leg is calculated by the ratio of the ankle over the higher brachial systolic blood pressure 3. ABI < 0.9 is not only established as a diagnostic method for PAD, but also a strong predictor for cardiovascular (CV) and all-cause mortality 4-6. In addition, ABI > 1.3 is also considered as abnormal and suggests hardening or calcification of the incompressible vessel walls, which reflects another form of PAD 7-10.

CHADS_2_, R_2_CHADS_2_, and CHA_2_DS_2_-VASc score are all useful scoring systems to evaluate the risk of stroke in patients with atrial fibrillation (AF) 11-16. In AF patients, there is a significant association between the CHADS_2_, R_2_CHADS_2_, and CHA_2_DS_2_-VASc score and the annual risk of stroke. Furthermore, these scoring systems were recently used to predict CV outcomes in the patients without AF 17-25. However, there was no literature discussing about the usefulness of CHADS_2_, R_2_CHADS_2_ and CHA_2_DS_2_-VASc score for CV and all-cause mortality prediction in the patients with abnormal low and high ABI. Therefore, we conducted this study to evaluate the issue.

## Materials and Methods

### Study population

Study population was enrolled from a group of patients arranged for examinations of echocardiography at Kaohsiung Municipal Siaogang Hospital from March 2010 to March 2012 because of suspecting hypertension, coronary artery disease, heart failure, and abnormal cardiac physical examination, and survey for dyspnea. Patients with abnormal low and high ABI defined as ABI < 0.9 or ABI > 1.3 were included. We excluded the subjects with significant atrial fibrillation, diseases of mitral and aortic valve. Finally, 195 patients were included in our study.

### Ethics Statement

Our study protocol was approved by the institutional review board committee of the Kaohsiung Medical University Hospital (KMUH-IRB). Informed consents have obtained from the patients and our study was conducted according to the principles expressed in the Declaration of Helsinki.

### Assessment of ABI

The values of ABI were measured by using an ABI-form device (VP1000; Colin Co. Ltd., Komaki, Japan), which automatically and simultaneously measures blood pressures in both arms and ankles using an oscillometric method 26. ABI of each leg was calculated by the ratio of the ankle over the higher brachial systolic blood pressure. After obtaining bilateral ABIs, the lower one was selected for later analysis.

### Calculation of CHADS_2_, R_2_CHADS_2_, and CHA_2_DS_2_-VASc score

CHADS_2_ score was calculated for each patient based on a scoring system in which 2 points were assigned for a history of stroke or transient ischemic attack and 1 point was assigned for age ≥75 years, the presence of hypertension, diabetes mellitus, and congestive heart failure 11,12. R_2_CHADS_2_ score was calculated for each patient based on a point system in which 2 points were assigned for renal dysfunction implicated by estimated glomerular filtration rate < 60 ml/min/1.73 m^2^ and a history of stroke, and 1 point was assigned for age ≥75 years, the presence of congestive heart failure, hypertension, and diabetes 16. CHA_2_DS_2_-VASc score was calculated for each patient based on a point system in which 2 points were assigned for age ≥75 years and a history of stroke, and 1 point was assigned for congestive heart failure, hypertension, age between 65 and 74 years, diabetes mellitus, female sex, and vascular disease 13.

### Collection of medical and demographic data

Medical data and demographic including age, gender, and other comorbid diseases were collected from medical records or interviews with patients. Hypertension was defined as systolic blood pressure ≥140 mmHg or diastolic blood pressure ≥90 mmHg or anti-hypertensive drugs were prescribed. Diabetes was defined as fasting blood glucose level greater than 126 mg/dL or hypoglycemic agents were used to control blood glucose levels. Dyslipidemia was defined as total cholesterol > 200 mg/dL, low density lipoprotein > 130 mg/dL, or triglycerides > 200 mg/dL. Information about patient medications including aspirin, angiotensin converting enzyme inhibitors, angiotensin II receptor blockers, β-blockers, calcium channel blockers, diuretics, and statin at enrollment was obtained from medical records.

### Definition of CV and all-cause mortality

All study participants were followed up till December 2018. Survival information and causes of death were obtained from the official death certificate and final confirmation by the Ministry of Health and Welfare. The causes of death were classified by the International Classification of Diseases 9^th^ Revision. Causes of CV mortality were defined deaths due to cerebral vascular disease, ischemic heart disease, myocardial infarction, heart failure, valvular heart disease and atherosclerotic vascular disease.

### Statistical analysis

We used SPSS 22.0 software for statistical analysis. Our data was expressed as mean ± standard deviation, percentage, or median (25th-75th percentile) for follow-up period. Continuous and categorical variables between groups were compared by independent samples *t* test and Chi-square test, respectively. The significant variables in the univariate analysis were selected for multivariable analysis. Time to the CV and overall mortality events and covariates of risk factors were modeled using the Cox proportional hazards model with forward selection. The incremental value of CHADS_2_, R_2_CHADS_2_, and CHA_2_DS_2_-VASc score over conventional parameters to assess the risk for CV and all-cause mortality were studied by calculating the improvement in global chi-square. Kaplan-Meier survival plots were calculated from baseline to time of mortality events. All tests were 2-sided and the level of significance was established as *P* < 0.05.

## Results

Among the 195 subjects, mean age was 66 ± 15 years. CHADS_2_, R_2_CHADS_2_, and CHA_2_DS_2_-VASc score in patients with ABI < 0.9 (n = 81) were 2.64±1.29, 4.12±1.82, and 4.27±1.60, respectively. CHADS_2_, R_2_CHADS_2_, and CHA_2_DS_2_-VASc score in patients with ABI > 1.3 (n = 114) were 1.42±1.03, 2.23±1.63, and 2.30±1.50, respectively. CV and overall mortality data were collected up to December 2018. Mortality data were obtained from the Collaboration Center of Health Information Application (CCHIA), Ministry of Health and Welfare, Executive Yuan, Taiwan. The follow-up period to mortality events was 90 (25th-75th percentile: 39-101) months in all patients. Mortality events were documented during the follow-up period, including CV mortality (n= 41) and all-cause mortality (n= 96).

**Table 1** compares the clinical characteristics between patients with and without mortality. Compared to patients without mortality, patients with mortality were found to have an older age, higher prevalence of diabetes mellitus, higher heart rate, lower body mass index (BMI), lower estimated glomerular filtration rate, higher CHADS_2_, R_2_CHADS_2_, and CHA_2_DS_2_-VASc score, and higher percentage of diuretic use.

**Table 2** shows the predictors of CV and all-cause mortality using Cox proportional hazards model by univariate analysis. Age, hypertension, diabetes, and renal function were not included in the univariate analysis because these variables were included in the three scoring systems. For prediction of CV mortality, higher mean blood pressure, increased heart rate, lower BMI, and higher CHADS_2_, R_2_CHADS_2_, and CHA_2_DS_2_-VASc score (all three *P* < 0.001) are significant predictors. For prediction of all-cause mortality, higher mean blood pressure, increased heart rate, lower BMI, higher CHADS_2_, R_2_CHADS_2_, and CHA_2_DS_2_-VASc score (all *P* < 0.001), and diuretic use are significant predictors.

**Table 3** shows the predictors of CV mortality using Cox proportional hazards model by multivariate analysis. All of the three models included the significant variables in the univariate analysis including mean blood pressure, heart rate, and BMI. CHADS_2_, R_2_CHADS_2_, and CHA_2_DS_2_-VASc score were added in model 1 to model 3, respectively. In model 1, higher mean blood pressure, increased heart rate, and higher CHADS_2_ score (hazard ratio [HR] = 1.632; 95% confidence interval [CI]: 1.252-2.129; *P* < 0.001) were significant predictors after multivariate analysis. In model 2, higher mean blood pressure, increased heart rate, lower BMI, and higher R_2_CHADS_2_ score (HR = 1.433; 95% CI: 1.181-1.739; *P* < 0.001) were significant predictors after multivariate analysis. In model 3, higher mean blood pressure, increased heart rate, and higher CHA_2_DS_2_-VASc score (HR = 1.639; 95% CI: 1.324-2.028; *P* < 0.001) were significant predictors after multivariate analysis.

**Table 4** shows the predictors of all-cause mortality using Cox proportional hazards model by multivariate analysis. All of the three models included the significant variables in the univariate analysis including mean blood pressure, heart rate, BMI, and diuretic use. CHADS_2_, R_2_CHADS_2_, and CHA_2_DS_2_-VASc score were added in model 1 to model 3, respectively. In model 1, higher mean blood pressure, lower BMI, and higher CHADS_2_ score (HR = 1.512; 95% CI: 1.268-1.802; *P* < 0.001) were significant predictors after multivariate analysis. In model 2, lower BMI, and higher R_2_CHADS_2_ score (HR = 1.410; 95% CI: 1.238-1.606; *P* < 0.001) were significant predictors after multivariate analysis. In model 3, higher mean blood pressure, increased heart rate, and higher CHA_2_DS_2_-VASc score (HR = 1.480; 95% CI: 1.290-1.699; *P* < 0.001) were significant predictors after multivariate analysis.

**Figure 1:**
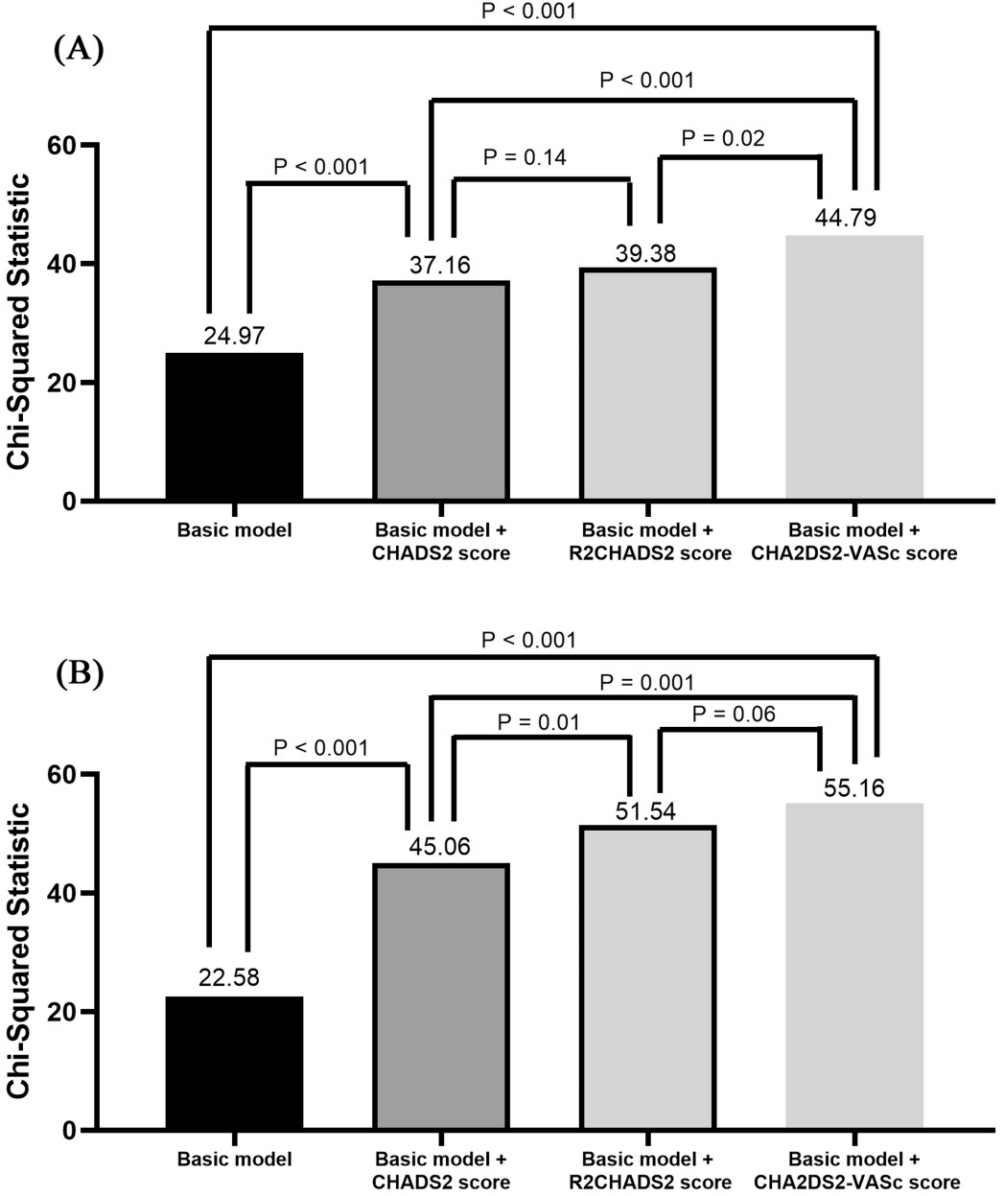
Nested Cox model for CV mortality (**Figure 1A**) and all-cause mortality (**Figure 1B**). Basic model in Figure 1A: mean blood pressure, heart rate, and body mass index. Basic model in Figure 1B: mean blood pressure, heart rate, body mass index, and diuretic use.

**Figure 1** shows the Nested Cox model for CV mortality (**Figure 1A**) and all-cause mortality (**Figure 1B**). The basic model in** Figure 1A** were mean blood pressure, heart rate, and body mass index. After adding CHADS_2_, R_2_CHADS_2_, and CHA_2_DS_2_-VASc score into the basic model respectively, we found these three models had better predictive values for CV mortality than basic model itself (all three *P* < 0.001). In addition, basic model + CHA_2_DS_2_-VASc score had a better predictive value for CV mortality than basic model + CHADS_2_ score and basic model + R_2_CHADS_2_ score (*P* = 0.005 and *P* = 0.02, respectively). The basic model in** Figure 1B** were mean blood pressure, heart rate, body mass index, and diuretic use. After adding CHADS_2_, R_2_CHADS_2_, and CHA_2_DS_2_-VASc score into the basic model respectively, we found these three models had better predictive values for all-cause mortality than basic model itself (all three *P* < 0.001). In addition, basic model + R_2_CHADS_2_ score and basic model + CHA_2_DS_2_-VASc score had better predictive values for all-cause mortality than basic model + CHADS_2_ score (*P* = 0.01 and *P* = 0.001, respectively).

**Figure 2:**
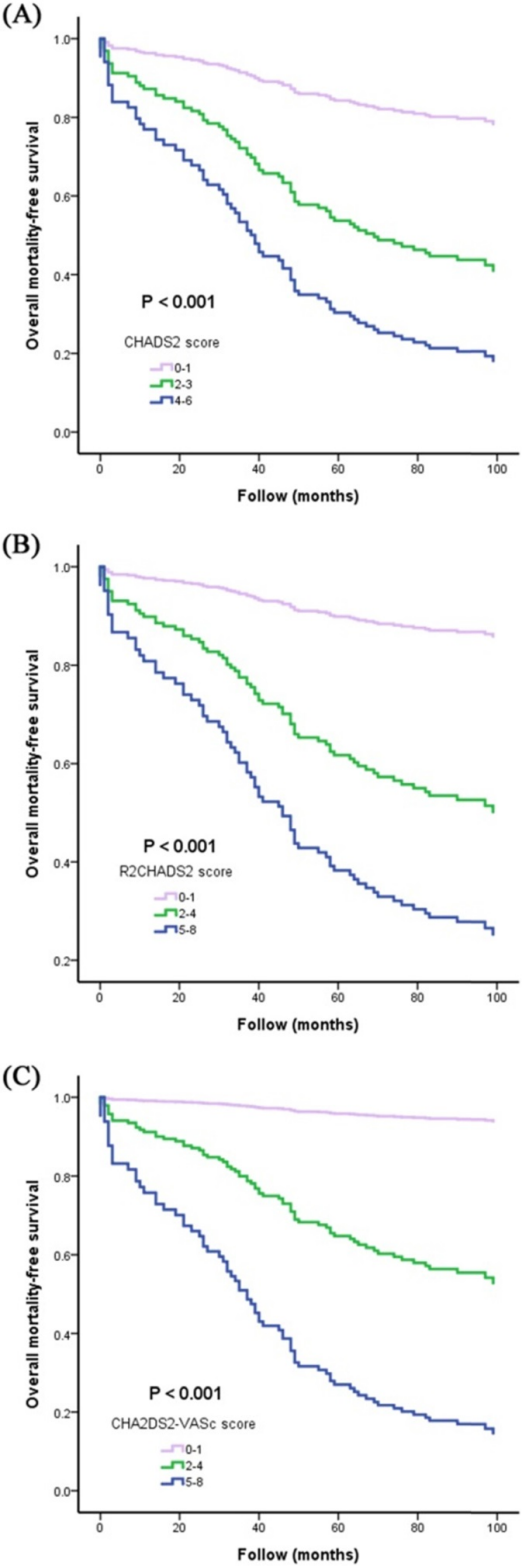
The Kaplan-Meier curves of CHADS_2_, R_2_CHADS_2_, and CHA_2_DS_2_-VASc score for all-cause mortality-free survival (**Figure 2A, 2B, and 2C**).

**Figure 2** illustrates the Kaplan-Meier curves of CHADS_2_, R_2_CHADS_2_, and CHA_2_DS_2_-VASc score for all-cause mortality-free survival (all three *P* < 0.001).

## Discussion

Our study was aimed to investigate the usefulness of CHADS_2_, R_2_CHADS_2_, and CHA_2_DS_2_-VASc score on the prediction of CV and all-cause mortality in patients with abnormal low and high ABI. There were several major findings in this study. First, CHADS_2_, R_2_CHADS_2_, and CHA_2_DS_2_-VASc score were significant predictors of CV and all-cause mortality after multivariate analysis. Second, CHADS_2_, R_2_CHADS_2_, and CHA_2_DS_2_-VASc score had additive predictive values than conventional parameters for prediction of CV and all-cause mortality. Third, basic model + CHA_2_DS_2_-VASc score had a better predictive value for CV mortality than basic model + CHADS_2_ score and basic model + R_2_CHADS_2_ score. Fourth, basic model + R_2_CHADS_2_ score and basic model + CHA_2_DS_2_-VASc score had better predictive values for all-cause mortality than basic model + CHADS_2_ score.

Abnormal low and high ABI are not only regarded as diagnostic parameters of PAD, but also strong predictors of CV and all-cause mortality 4-10. PAD is a systemic atherosclerotic disease with similar risk factors as cerebrovascular disease and coronary artery disease (CAD) 27. Risk factors for PAD include advanced age, hypertension, diabetes mellitus, dyslipidemia, cigarette smoking, races, chronic kidney disease, and so on 27,28. Patients with PAD had extremely high morbidity and mortality 29,30. 5-year mortality of patients with symptomatic and asymptomatic PAOD is 24% and 19% 29. However, mortality could be as high as 20% within 6 months from diagnosis and exceeding 50% at 5 years for the patients with critical limb ischemia 30. Therefore, how to identify high risk PAD patients with increased mortality become extremely important.

CHADS_2_, R_2_CHADS_2_, and CHA_2_DS_2_-VASc score are all simple and useful scoring system to evaluate the risk of stroke in AF patients 11-16. However, R_2_CHADS_2_ score and CHA_2_DS_2_-VASc score have recently become more useful scores and outperformed CHADS_2_ score for prediction of stroke and systemic embolization 13,16. In addition, all three scores were used to predict CV outcomes and mortality in non-AF patients 17-25. Li Y et al. used CHADS_2_ and R_2_CHADS_2_ score to predict long-term outcome for CAD patients and their data showed both of scores are useful tools in predicting long-term outcome 22. Hoshino T et al. investigated CHADS_2_, R_2_CHADS_2_, and CHA_2_DS_2_-VASc score for prediction of 3-month functional outcome of stroke in patients with prior CAD and they found all three scores are useful scoring systems for predicting the outcomes 21. Chen YL et al. evaluated CHADS_2_, R_2_CHADS_2_, and CHA_2_DS_2_-VASc scores for prediction of 1-year all-cause mortality for patients with systolic heart failure and results showed all three scores can be used to predict the mortality; however, R_2_CHADS_2_ score is more accurate than CHADS_2_ and CHA_2_DS_2_-VASc score 20. Furthermore, these three scoring systems were also reported to be useful tools for predicting outcomes in patients with acute coronary syndrome, syncope, sick sinus syndrome, heart failure, and so on 19,23-25. However, there was no literature investigating the usefulness of CHADS_2_, R_2_CHADS_2_, and CHA_2_DS_2_-VASc score for CV and all-cause mortality prediction in the patients with abnormal low and high ABI. Our study is the first study to discuss the issue.

In our study, all of CHADS_2_, R_2_CHADS_2_, and CHA_2_DS_2_-VASc score were associated with increased CV and all-cause mortality in univariate and multivariate analysis. In addition, although all three scores had additive predictive values than conventional parameters for prediction of CV and all-cause mortality, CHA_2_DS_2_-VASc score had a better predictive value than CHADS_2_ and R_2_CHADS_2_ score for prediction of CV mortality in direct comparison of multivariate model (*P* = 0.005 and 0.02, respectively). R_2_CHADS_2_ and CHA_2_DS_2_-VASc score also had better predictive values than CHADS_2_ score for prediction of all-cause mortality in direct comparison of multivariate model (*P* = 0.01 and 0.001, respectively).

## Study Limitations

First, our study was aimed to investigate the mortality outcome, so non-fatal outcomes were not evaluated. Second, the majority of our patients were treated with CV medications. For ethical reasons, we did not withdraw these medications. Hence, we could not exclude the influence of CV medications on our study. However, we already adjusted the associated usage of CV medications in multivariate analysis, which can reduce the influence of medication.

## Conclusions

Our study is the first study to investigate the usefulness of CHADS_2_, R_2_CHADS_2_, and CHA_2_DS_2_-VASc score in patients with abnormal low and high ABI for prediction of long-term CV and all-cause mortality. Our study showed all three scores are significant predictors for long-term CV and all-cause mortality. In addition, CHA_2_DS_2_-VASc score had a better predictive value than CHADS_2_ and R_2_CHADS_2_ score for CV mortality, and R_2_CHADS_2_ and CHA_2_DS_2_-VASc score had better predictive values than CHADS_2_ score for all-cause mortality in direct comparison of multivariate model. Therefore, using CHADS_2_, R_2_CHADS_2_, and CHA_2_DS_2_-VASc score to screen patients with abnormal low and high ABI can help physicians to identify the high-risk PAD patients with increased mortality.

## Figures and Tables

**Table 1:** Comparison of clinical characteristics between patients with mortality and without mortality

Baseline Characteristics	Mortality (-)	Mortality (+)	*P* value
Number	99	96	
Age (yr)	60 ± 13	73 ± 13	<0.001
Male gender (%)	71.7%	59.4%	0.073
Smoking (%)	15.2%	8.3%	0.183
Diabetes (%)	32.3%	51.0%	0.009
Hypertension (%)	78.6%	85.4%	0.264
Dyslipidemia (%)	49.3%	42.1%	0.475
Heart rate (min^-1^)	68 ± 12	72 ± 13	0.013
Body mass index (kg/m^2^)	27.2 ± 4.4	25.4 ± 4.4	0.005
eGFR	59.5 ± 17.6	40.8 ± 20.3	<0.001
CHADS_2_ score	1.38 ± 1.04	2.49 ± 1.29	<0.001
R_2_CHADS_2_ score	2.13 ± 1.65	3.93 ± 1.81	<0.001
CHA_2_DS_2_-VASc score	2.29 ± 1.51	3.97 ± 1.73	<0.001
**Medication**			
Aspirin	43.3%	46.1%	0.756
β-blockers	45.5%	35.4%	0.189
CCBs	42.4%	51.0%	0.252
ACEIs	9.1%	16.8%	0.134
ARBs	49.5%	57.3%	0.316
Diuretics	30.3%	52.1%	0.002
Statin	45.5%	40.6%	0.563

Abbreviations: ACEI, angiotensin converting enzyme inhibitor; ARB, angiotensin II receptor blocker; CCB, calcium channel blocker; eGFR, estimated glomerular filtration rate.

**Table 2:** Predictors of cardiovascular events (all-cause mortality) using Cox proportional hazards model

Parameter	Univariate (CV mortality)		Univariate (all-cause mortality)	
	HR (95% CI)	*P*	HR (95% CI)	*P*
Male gender	0.815 (0.398-1.668)	0.576	0.691 (0.432-1.104)	0.122
Mean blood pressure (mmHg)	1.039 (1.015-1.062)	0.001	1.019 (1.003-1.036)	0.018
Dyslipidemia (Yes or No)	1.421 (0.599-3.373)	0.426	0.772 (0.452-1.320)	0.344
Smoking (ever vs no)	0.836 (0.293-2.386)	0.738	0.724 (0.347-1.510)	0.389
Heart rate (per beat/minute)	1.039 (1.015-1.062)	0.001	1.020 (1.003-1.039)	0.023
Body mass index (per kg/m^2^)	0.894 (0.815-0.981)	0.017	0.928 (0.876-0.984)	0.012
CHADS_2_ score	1.643 (1.295-2.085)	<0.001	1.580 (1.347-1.854)	<0.001
R_2_CHADS_2_ score	1.479 (1.237-1.769)	<0.001	1.449 (1.286-1.632)	<0.001
CHA_2_DS_2_-VASc score	1.578 (1.308-1.903)	<0.001	1.502 (1.326-1.702)	<0.001
**Medications**				
Aspirin use	0.658 (0.312-1.391)	0.273	1.088 (0.682-1.735)	0.724
Beta blocker use	1.074 (0.534-2.160)	0.841	0.733 (0.451-1.192)	0.211
Calcium channel blocker use	1.329 (0.660-2.676)	0.425	1.274 (0.800-2.029)	0.308
ACEI use	1.192 (0.417-3.409)	0.743	1.151 (0.572-2.315)	0.694
ARB use	1.131 (0.562-2.276)	0.730	1.368 (0.851-2.198)	0.196
Diuretic use	1.954 (0.971-3.934)	0.061	1.872 (1.175-2.982)	0.008

HR: hazard ratio; CI: confidence interval; other abbreviations as in Table 1.

**Table 3:** Predictors of CV mortality using Cox proportional hazards model (multivariate analysis)

Parameter	Model 1	*P*	Model 2	*P*	Model 3	*P*
	HR (95% CI)		HR (95% CI)		HR (95% CI)	
MBP (mmHg)	1.038 (1.03-1.063)	0.003	1.032 (1.007-1.059)	0.013	1.043 (1.018-1.068)	0.001
HR (beat/min)	1.043 (1.015-1.072)	0.003	1.041 (1.012-1.070)	0.005	1.050 (1.019-1.082)	0.001
BMI (kg/m^2^)	0.913 (0.831-1.002)	0.056	0.910 (0.829-0.999)	0.047	0.925 (0.841-1.017)	0.107
CHADS_2_ score	1.632 (1.252-2.129)	<0.001	-	-	-	-
R_2_CHADS_2_ score	-	-	1.433 (1.181-1.739)	<0.001	-	-
CHA_2_DS_2_-VASc score	-	-	-	-	1.639 (1.324-2.028)	<0.001

HR: hazard ratio; CI: confidence interval; other abbreviations as in Table 1.

**Table 4:** Predictors of overall mortality using Cox proportional hazards model (multivariate analysis)

Parameter	Model 1	*P*	Model 2	*P*	Model 3	*P*
	HR (95% CI)		HR (95% CI)		HR (95% CI)	
MBP (mmHg)	1.019 (1.002-1.036)	0.031	1.014 (0.996-1.032)	0.119	1.023 (1.005-1.040)	0.010
HR (beat/min)	1.018 (0.998-1.039)	0.084	1.019 (0.998-1.040)	0.070	1.020 (0.999-1.043)	0.010
BMI (kg/m^2^)	0.941 (0.886-0.999)	0.045	0.940 (0.886-0.997)	0.040	0.953 (0.897-1.012)	0.114
Diuretic	1.246 (0.750-2.071)	0.396	1.079 (0.643-1.813)	0.773	1.150 (0.694-1.907)	0.587
CHADS_2_ score	1.512 (1.268-1.802)	<0.001	-	-	-	-
R_2_CHADS_2_ score	-	-	1.410 (1.238-1.606)	<0.001	-	-
CHA_2_DS_2_-VASc score	-	-	-	-	1.480 (1.290-1.699)	<0.001

HR: hazard ratio; CI: confidence interval; other abbreviations as in Table 1.
